# Endovascular Embolization of Pulmonary Sequestration Presenting With Hemoptysis: A Promising Alternative to Surgery

**DOI:** 10.7759/cureus.17399

**Published:** 2021-08-23

**Authors:** Sherif Roman, Christopher Millet, Nader Mekheal, Erinie Mekheal, Rajapriya Manickam

**Affiliations:** 1 Internal Medicine, St. Joseph's Regional Medical Center, Paterson, USA; 2 Pulmonary and Critical Care Medicine, St. Joseph's Regional Medical Center, Paterson, USA

**Keywords:** intralobar, sequestration, coils, embolization, recurrent pneumonia, hemoptysis, ct angiography

## Abstract

Pulmonary sequestration is abnormal lung parenchyma separated from the normal tracheobronchial system and supplied by an aberrant systemic artery. It is usually asymptomatic; however, it can present with hemoptysis and recurrent pulmonary infections. Although surgery is the classical treatment, arterial embolization is recently established as an alternative treatment to avoid surgical complications. We present a case of left lower lobe intralobar pulmonary sequestration presented with hemoptysis and was successfully treated with coil embolization.

## Introduction

Pulmonary sequestration is a dysplastic, nonfunctioning lung tissue supplied by systemic blood circulation. It is a rare disease accounting for 0.15%-6.4% of congenital pulmonary anomalies [1]. It has two types - either intralobar sequestration (ILS) or extralobar sequestration (ELS). The condition is asymptomatic in most cases; however, it can also cause recurrent infections and hemoptysis [2].

The traditional treatment is surgical resection of the lung sequestration, but arterial embolization has been introduced as an alternative treatment in the last few years. We report a case of intralobar pulmonary sequestration presented with hemoptysis. It was successfully treated with coil embolization of two branches of the distal thoracic aorta that were supplying the sequestered lung.

## Case presentation

A 51-year-old Filipino male presented to our facility complaining of hemoptysis for three days. The patient stated that he coughs up streaks of dark blood, about 1/2 teaspoon occasionally when coughing, and quantifies approximately two teaspoons per day of blood. The patient also reports exertional shortness of breath, but he denies chest pain, fever, nausea, vomiting, night sweats, or weight loss. He also denied any history of smoking or blood thinners intake.

The patient works as a telemetry nurse and he had a mild COVID-19 infection three weeks ago. He added that his symptoms resolved and the repeat COVID-19 test was negative. Initial vital signs showed a temperature of 36.7 ºC, heart rate 123/min, blood pressure 133/96, respiratory rate of 18/min, and SpO_2_ of 98% on room air.

**Figure 1:**
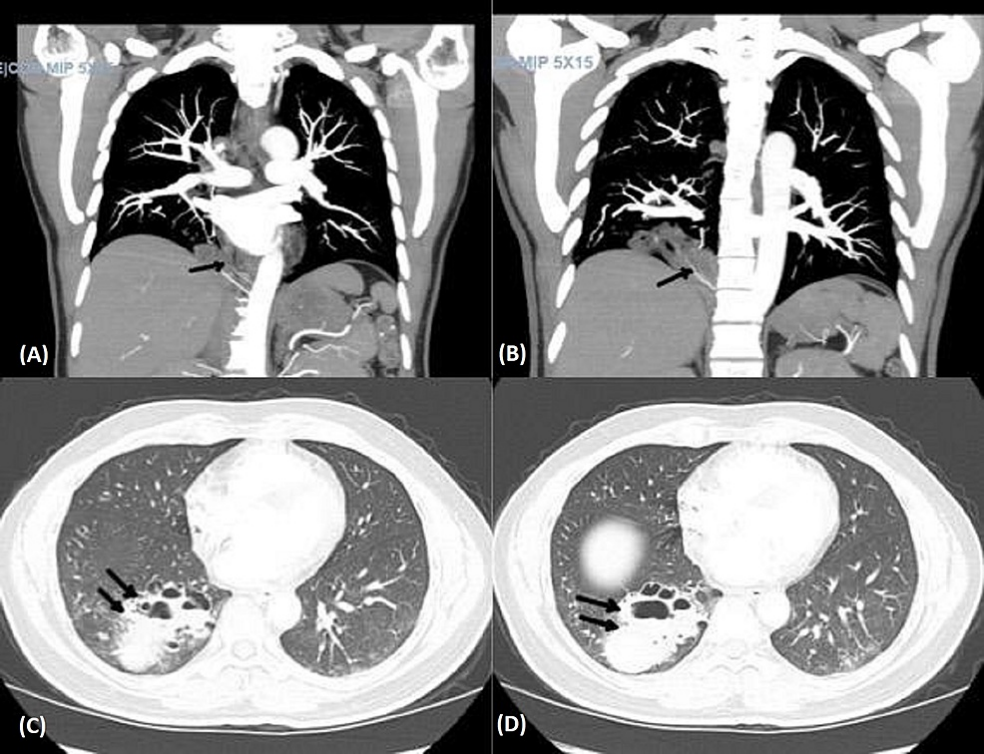
Computed tomography (CT) angiography of the chest showing intralobar pulmonary sequestration at the right lung base with aberrant feeding arteries arising from the lower thoracic aorta (A,B), which is likely the source of acute blood products within this complex blood-filled cavity (C,D).

The lung exam was significant for decreased air entry with rales and rhonchi heard on the right lower lobe. Laboratory analysis revealed a leukocytosis of 14,800 with no bandemia. Workup for infection, including SARS-CoV-2, respiratory viral panel, blood, and sputum cultures, were remarkable for the growth of Haemophilus parainfluenzae on sputum culture. A plain chest radiograph demonstrated no focal consolidation or pleural effusion. The patient was started on ceftriaxone and azithromycin. To rule out pulmonary embolism as a cause of hemoptysis and tachycardia, Computed tomography (CT) angiography of the chest was done and showed findings compatible with intralobar pulmonary sequestration at the right lung base with aberrant feeding arteries arising from the lower thoracic aorta (Figures 1A, 1B), which is likely the source of acute blood products within this complex blood-filled cavity (Figures 1C, 1D).

Treatment options, including surgical resection and arterial embolization, were explained in full detail to the patient. He chose to proceed with arterial embolization. Under local anesthesia, access was gained through the right common femoral artery.

**Figure 2:**
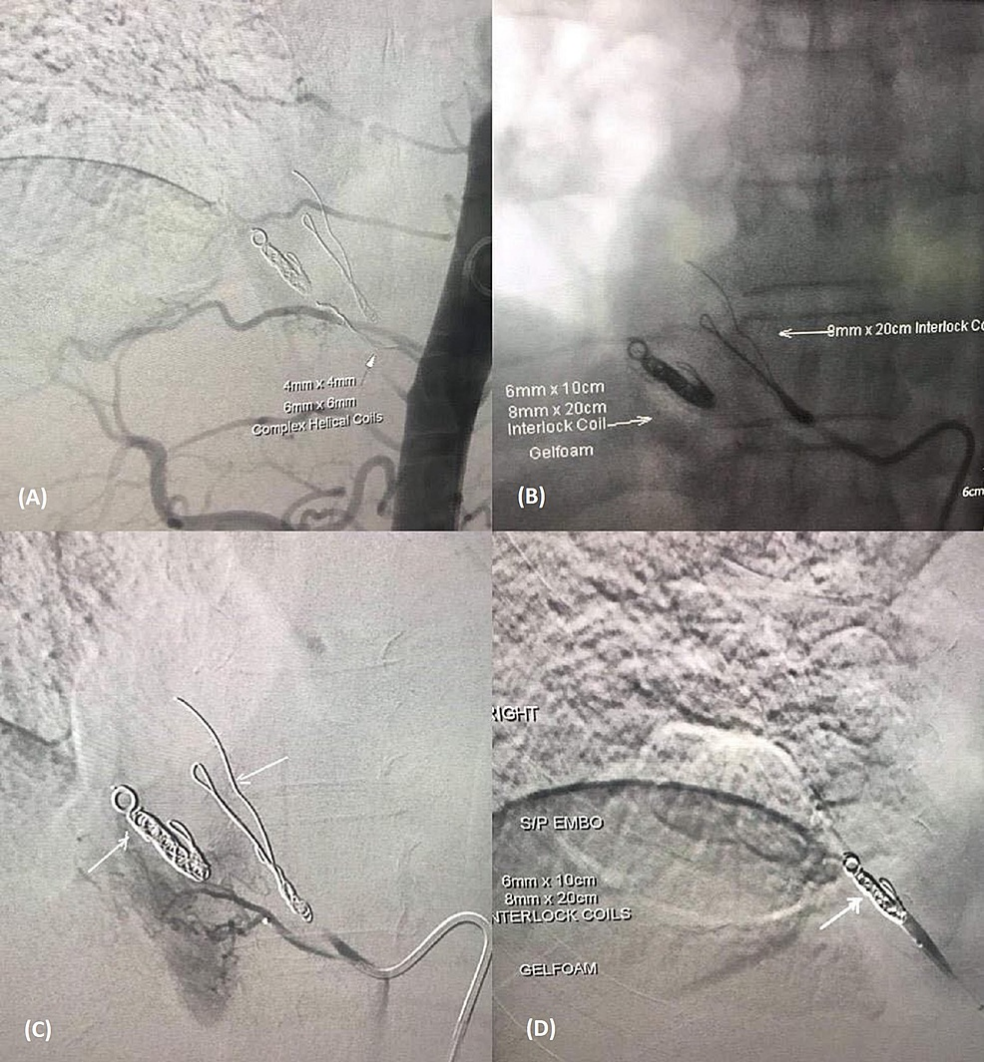
Endovascular coil embolization of two systemic arteries originating from the descending thoracic aorta supplying intrapulmonary sequestration in right lower lobe with good angiographic results (A-D).

A catheter was then advanced into the lower thoracic aorta and an angiogram was performed. The branch supplying the right lower lobe pulmonary sequestration was embolized with 6 mm and 8 mm coils. Just superiorly, there was another smaller branch supplying the pulmonary sequestration from the thoracic aorta. It was also embolized with 4 mm and 5 mm coils. Repeat angiogram demonstrates no perfusion of the pulmonary sequestration (Figures 2A-2D). 

A significant improvement in the patient's condition was noted post embolization. His symptoms have been resolved, and his hemoglobin level remained stable. After spending four days in the hospital, he was discharged home and was instructed to follow up in the outpatient clinic.

## Discussion

Pulmonary sequestration is a nonfunctional parenchymal lung tissue unconnected to the tracheobronchial system and receives systemic blood supply rather than the normal pulmonary arterial supply [3]. The condition was first described by Pryce, who classified it into intralobar and extralobar types [4].

The common type is the ILS (about 75%), where the dysplastic parenchyma is surrounded by normal lung tissue and commonly involves the lower pulmonary lobes. It is usually located on the left side; however, it involved the right side in our case. The other type is the ELS (about 25%), which has its pleural investment [5].

Pulmonary sequestrations are often asymptomatic but they may present with cough, fever, chest pain, recurrent pulmonary infections, and hemoptysis [6]. In our case, hemoptysis was the prominent symptom.

The pathogenesis of pulmonary sequestration is uncertain and multiple hypotheses have been proposed to explain it. Infections may have a role in pathogenesis; however, congenital reasons cannot be omitted. It is theorized that the sequestration is a remnant of the ventral foregut, which migrates caudally [6].

The ILSs receive their systemic arterial supply via the descending thoracic aorta (72%), abdominal aorta (21%), and the intercostal arteries (3%) [7]. In the present case, the feeding branches originated from the distal thoracic aorta. The diagnosis of pulmonary sequestration is generally based on imaging and recognizing the systemic blood supply. CT angiography can identify the origin and course of the aberrant systemic artery.

Surgical resection of the pulmonary sequestration is the classical therapeutic approach to prevent massive hemoptysis, recurrent pulmonary infections, or other symptoms. Its benefit is still questionable in patients with no symptoms [8]. Resection can be performed with either video-assisted thoracoscopic procedures or open thoracotomy; however, these procedures may have complications. In a retrospective study, 28% of patients with ILS and ELS had post-procedure complications, including bronchopulmonary fistula, bleeding, chest pain, and pneumonia [9].

Arterial embolization is recently established as an alternative treatment to avoid these complications. Embolization occludes the anomalous arterial supply, causing involution of the sequestration by infarction, necrosis, and fibrosis [5]. It is a promising option in pulmonary sequestration treatment as it reduces complications and shortens hospital stays.

Despite the advantages of embolization, it can lead to complications such as access site thrombosis, nontarget embolization, and transient limb ischemia [10]. Recurrence of the sequestration after embolization may occur due to the creation of shunts, incomplete closure, and embolization of non-targeted arteries. More research about the long-term outcomes of patients undergoing sequestration embolization is needed to better evaluate this alternative treatment modality.

## Conclusions

Pulmonary sequestration is a rare condition that can present with hemoptysis and recurrent pulmonary infections. CT angiography can identify the aberrant systemic artery supplying the sequestered lung. Surgical resection is the classical treatment; however, arterial embolization is recently established as an alternative treatment to avoid surgical complications. Further studies are necessary to determine the long-term outcomes of this procedure.
